# Legacy Effects of Flooding Duration on Growth and Reproductive Traits of *Carex cinerascens* in the Poyang Lake Wetland

**DOI:** 10.1002/ece3.71395

**Published:** 2025-05-06

**Authors:** Wenlan Feng, Pierre Mariotte, Ligang Xu, Luca Bragazza, Alexandre Buttler, Junxiang Cheng, Mathieu Santonja

**Affiliations:** ^1^ National key Laboratory of Lake and Watershed Science for Water Security Nanjing Institute of Geography and Limnology, Chinese Academy of Sciences Nanjing China; ^2^ State Key Laboratory of Soil and Sustainable Agriculture Institute of Soil Science, Chinese Academy of Sciences Nanjing China; ^3^ Ecole Polytechnique Fédérale de Lausanne (EPFL), School of Architecture, Civil and Environmental Engineering (ENAC) Laboratory of Ecological Systems (ECOS) Lausanne Switzerland; ^4^ Swiss Federal Institute for Forest, Snow and Landscape Research (WSL) Lausanne Switzerland; ^5^ Agroscope Grazing Systems Fribourg Switzerland; ^6^ University of Chinese Academy of Sciences Beijing China; ^7^ Agroscope Field‐Crop Systems and Plant Nutrition Nyon Switzerland; ^8^ Aix Marseille Univ, Avignon Univ, CNRS, IRD IMBE Marseille France

**Keywords:** flood regime, hydrodynamics, plant biomass, sexual reproduction, wetlands

## Abstract

Alteration of flooding regimes due to global change may have cascading effects on plant community composition and associated ecosystem services. Here, we experimentally investigated the effects of six flooding regimes with contrasting combinations of flooding duration (5.5, 6 and 6.5 months) and submergence rate (from 3.3 to 17.5 cm/day) on the growth and reproductive traits of *Carex cinerascens*, a dominant plant species of the Poyang Lake wetland in southern China. The time span of this study included a summer flooding event and the following growing seasons (autumn of first year and spring of following year) before the return of the next flooding event. The six flooding treatments affected plant traits during the flooding and the following growing seasons, but the different submergence rates under the same flooding duration did generally not show significant influence on plant traits. The 6.5‐month flooding treatments had many fewer old (0.4 on average) and new stems (1 on average) than the 5.5‐month treatments (8.3 and 29 stems, respectively) at the end of the flooding. The treatments with 5.5 months of flooding had 23% more stems than the other treatments and 26% more community biomass than the 6‐month flooding treatments during the autumn growing season. The effects of summer flooding persisted in spring of the following year, but with an opposite trend of *C. cinerascens* growth traits response to flooding treatments compared to autumn. In addition, the 6‐month flooding treatments induced a higher number of inflorescences (39) than the 5.5‐month (22) and 6.5‐month floods (3). Altogether, our findings highlighted the important legacy effects of summer flooding with some trade‐offs between growth recovery (autumn) and resilience (following spring) and between resource allocation to biomass production in autumn and resource allocation to sexual reproduction in the following spring, that were both mediated by flooding duration.

## Graphical abstract

We experimentally investigated the effects of six flooding regimes with contrasting combinations of flooding duration and submergence rate on the growth and reproductive traits of *Carex cinerascens*, a dominant plant species of the Poyang Lake wetland in southern China. The effects observed on the growth and reproductive traits of *Carex cinerascens* were only dependent on flooding duration but not submergence rate. The effects of summer flooding persisted in spring of the following year, but with an opposite trend of *C. cinerascens* growth traits response to flooding treatments compared to autumn.

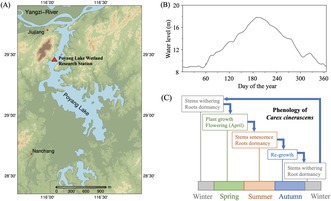

## 1 Introduction

Wetlands are important ecosystems that provide a wide variety of habitats for plants and animals (Dawson et al. 2003; Neckles et al. 2010), as well as several important ecosystem functions such as water purification, water storage, and shoreline stabilization (Dawson et al. 2003). Wetland plants are well adapted to the occurrence of seasonal flooding periods (Pezeshki 2001). However, climate change is expected to alter the frequency, duration, and severity of floods (Erwin and Gardner 2009; Langerwisch et al. 2013; Tan et al. 2015; Li et al. 2023) with associated impacts on vegetation through changes in the hydrological regime (Visser et al. 2000; Zedler 2010; Sarneel et al. 2019; Feng, Mariotte, et al. 2020). In addition, the intensification of anthropogenic activities, such as sand mining (Lai et al. 2014) and dam construction (Guo et al. 2012; Hu et al. 2018; Zhang et al. 2022; Baladron et al. 2023), is also altering lake discharge and river flow, resulting in strong fluctuations in hydrological conditions experienced by wetland ecosystems.

Several studies have reported that hydrological processes control the spatial and temporal heterogeneity of wetland vegetation and associated ecosystem functions (Renofalt et al. 2007; Webb et al. 2012; Garssen et al. 2015; Feng, Santonja, et al. 2020; Huang et al. 2022). Flooding duration has been shown to be one of the crucial hydrological processes driving plant growth, plant reproduction, and thus plant community structure (Chen and Xie 2009; Hu et al. 2015; Hu et al. 2022). Seed germination, plant survival, growth, and reproductive parameters can be reduced by flooding due to oxygen depletion (Bailey‐Serres and Voesenek 2008; Paillisson and Marion 2011; Pan et al. 2020), stable day and night temperatures (Altenfelder et al. 2016), decreased light availability (Clevering et al. 1996; Casanova and Brock 2000; Mommer and Visser 2005) and changes in soil properties (Chen et al. 2013). Longer and more intense flooding periods can reduce plant abundance (Campbell et al. 2016) and plant species richness (Casanova and Brock 2000; Raulings et al. 2010). For example, Campbell et al. (2016) observed a lack of plant survival when flooding occurred during the whole growing season in Louisiana (USA). Furthermore, Garssen et al. (2017) reported a decrease in plant species richness, but an increase in plant biomass under prolonged flooding duration during a 3‐year field experiment in Northwestern Europe. However, despite the fact that several studies have investigated the effects of a flooding event on the following plant growing season (e.g., Lunt et al. 2012; Campbell et al. 2016), the extent to which a summer flooding event could affect plant growth in different growing periods (autumn vs. spring) before the next flooding event remains poorly understood.

In addition, much less attention has been paid to the submergence rate, that is, the speed of water level rise, in combination with flooding duration. Submergence rate is a key factor driving aquatic plant fitness, as it affects plant metabolism and thus the rate of stem and leaf development (Cooling et al. 2001; Yu and Yu 2011; Voesenek et al. 2016; Yao et al. 2021). With increasing submergence rate, more biomass is allocated to stem and leaf growth in aquatic plants (Yang et al. 2004; Deegan et al. 2012; Wei et al. 2014). However, the response of semi‐terrestrial wetland plant species that experience varying submergence rates during flooding remains unclear. Thus, knowledge of plant community responses to flooding that varies in both duration and submergence rate is very limited.

Poyang Lake is the largest freshwater lake in China, which experiences large water level fluctuations under the monsoon climate (Lai et al. 2014; Li et al. 2023). The response of wetland plants to the water regime is thus an important research topic in Poyang Lake wetlands (e.g., Zhou et al. 2018; Huang et al. 2022; Zheng et al. 2021). Previous studies have shown that summer flooding events affect vegetation distribution, plant cover, and plant biomass in this wetland (You et al. 2015; Dai et al. 2020; Li et al. 2020) and that intense and prolonged flooding events in summer can inhibit plant recovery and reproduction (Cui et al. 2000). Controlled experiments have been conducted to determine the effects of flooding duration (Li et al. 2018; Liu et al. 2023) and submergence rate along with flooding depth (Yao et al. 2021) on the growth and reproduction of wetland plants during flooding events. These studies pointed out that longer flooding duration, greater flooding depth, and faster submergence rate prevent the growth of wetland plants. However, it is still unclear how the combination of flooding duration and submergence rate in summer affects plant growth and reproduction in the subsequent autumn and spring growing seasons.

To fill this knowledge gap, we investigated the effects of six flooding scenarios on the growth and reproductive traits of *Carex cinerascens* Kük., a widely distributed dominant plant species in the Poyang Lake wetlands. We hypothesized that: (i) increasing flooding duration and submergence rate will induce more negative effects on *C. cinerascens* growth traits during the summer flooding and the following autumn growing season; (ii) a faster submergence rate will amplify the effect of flooding duration on *C. cinerascens* growth traits; (iii) the effects of the flooding treatments will not persist after the autumn period and cannot be detected in the following spring.

## 2 Materials and Methods

### 2.1 Study Site

**FIGURE 1:**
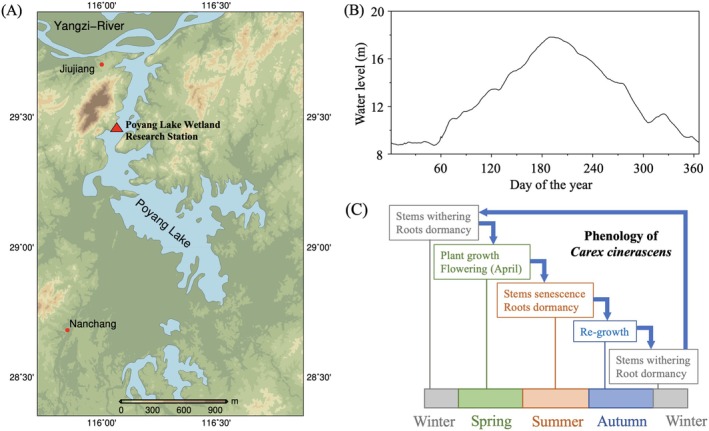
Map of the Poyang Lake with the location of the Research Station (red triangle) (A), mean water level of Poyang Lake (Xingzi Station, 2008–2017, in meter from Woosung Horizontal Zero) (B), and general plant phenology pattern of *C. cinerascens* (C).

The Poyang Lake (28.37°–29.75°N, 115.78°–116.75°E) is currently the largest freshwater lake in China. The climate is subtropical monsoon with a mean annual temperature of 17.6°C and mean annual precipitation of 1528 mm. The combined effects of catchment inflows and the interaction with the Yangtze River cause flooding in summer, resulting in large annual and seasonal variations in water level, which can reach up to 10 m (Zhang et al. 2014). In the Poyang Lake wetland, *Carex cinerascens* Kük. is the most widely distributed hygrophyte plant species with high productivity (You et al. 2015; Yuan et al. 2017). *Carex cinerascens‐*dominated communities are located close to the shoreline at an elevation of about 13–14 m above Woosung Horizontal Zero in China and are completely submerged in summer due to flooding that raises the water level above 14 m (Figure 1B), and to the low elevation gradient along the shore (Wang et al. 2016; Zhang et al. 2019).

### 2.2 Plant Material Sampling

The experiment was conducted at the Poyang Lake Wetland Research Station (29.45°N, 116.06°E, Figure 1A) where *C. cinerascens* emerges with increasing temperature in February, flowers and fruits in April, with biomass peaking at the end of April (Figure 1C). Plants then begin to senesce just before the summer flooding. During the flooding period, individuals are completely submerged and the aboveground part decays. While new stems continue to be produced at the beginning of the flooding, they quickly decay when the water level further increases. After the recession of flooding in autumn, the plants immediately sprout and regrow, yielding a second peak of biomass around November–December. The aboveground plant parts generally wither as temperature drops in winter, and new green stems sprout again in the following spring.

In April 2016 (i.e., before the flooding period), 36 turfs (30 cm diameter × 30 cm depth) were haphazardly collected from the wetland area near the Poyang Lake Wetland Research Station. The turf contained both intact *C. cinerascens* individuals and the corresponding undisturbed soil. Three soil samples were collected from the area where the turfs were collected in order to characterize the soil physico‐chemical characteristics. The soil collected from this area had on average a pH of 6.3, a dry bulk density of 1.21 g.cm^−3^, a total organic carbon concentration of 27.19 g.kg^−1^, a total nitrogen concentration of 1.43 g.kg^−1^, and a total phosphorus concentration of 0.86 g.kg^−1^. At the same time, the subsoil under the extracted turfs was collected to fill the bottom of the pots used in the experiment.

### 2.3 Experiment Design

The experiment was set up with six flooding scenarios, corresponding to different combinations of flooding duration and submergence rate. Since longer flooding durations are generally associated with higher rates of submergence (Figure S1), we adapted the submergence rate in function of the flooding duration, with higher submergence rates in longer flooding treatments. There were three flooding durations (5.5 months, 6 months and 6.5 months) each including two submergence rates (slower and faster), with the faster submergence rate defined as a 50% increase of the slower submergence rate. The submergence rate simulation was performed over a 3‐day period by adjusting the water level daily according to a specific schedule for each treatment (Table S1, Figure S2). The six flooding scenarios consisted of combinations of flooding duration (months) and submergence rate (average cm increase per day) and included 5.5 months with 3.3 cm/day, 5.5 months with 5 cm/day, 6 months with 6.7 cm/day, 6 months with 10 cm/day, 6.5 months with 11.7 cm/day, and 6.5 months with 17.5 cm/day, with 6 replicates per scenario.

The experiment was conducted in three tanks (2 × 2 × 1.7 m) from April 2016 to May 2017. The 36 turfs were placed in individual pots (30 cm diameter × 40 cm height) which were filled with 10 cm of subsoil at the bottom. Twelve pots were randomly assigned per tank, representing two replicates of each flooding scenario per tank. All pots had a similar number of stems at the beginning of the experiment before flooding (*F* = 0.05, *p* = 0.9981), with an average of 104 ± 10 individuals. The pots were suspended from a tube supported by a racket above the tank to manually control the submergence rate under a given flooding duration. The three tanks were filled with tap water instead of lake water in order to avoid confounding effects related to initial differences in water chemistry between tanks. One water sample was taken in each tank to characterize its nutrient concentrations. The tap water had, on average, a low nutrient concentration (0.16 mg. L^−1^ of N‐NH_4_^+^, 0.65 mg. L^−1^ of N‐NO_3_^−^, and 0.10 mg. L^−1^ of P‐PO_4_^3+^).

For an initial period of 3 days, the water level was kept 10 cm below the soil surface to acclimate the plants. The flooding simulation began on April 21, and the submergence depth was adjusted daily using the rope connecting the tube to the pot. After all pots of the fast submergence rate under each flooding duration touched the bottom of the tank (i.e., corresponding to 1.3 m of flooding depth), they remained in this position until the pots of the slow submergence rate under the same flooding duration also reached the same position. In the field, the *C. cinerascens* ‐dominated communities are sometimes flooded under a water column of more than 4 m, suggesting that there is almost no effective solar radiation for the submerged plants. To mimic this lack of radiation, all pots of the same flooding duration were covered with a sun shading net as soon as they reached the bottom of the tank (Figure S3a). Thus, within each flooding duration treatment, both submergence rate treatments experienced the same length of time without effective solar radiation. At the end of the flooding, the sun shading net was removed and the pots were lifted with the rope (on September 30, October 15 and October 30 for the 5.5, 6 and 6.5‐month flooding treatments, respectively), and kept at a water level of 10–20 cm below the soil surface until the end of the experiment (Figure S3).

### 2.4 Plant Trait Measurements

After 1 month of flooding, the number of new stems per pot and the height (cm) of five haphazardly selected new stems were measured. The number of both old and new stems per pot and the height (cm) of five haphazardly selected new stems were also measured at the end of each flooding period for each treatment. Old stems refer to stems that were present at the start of flooding, as indicated by their yellow color, while new stems refer to the regrowing *C. cinerascens* stems, as indicated by their green color.

At the peak of biomass production after the flooding event (autumn), on October 30, November 30, and December 30 for the 5.5, 6, and 6.5‐month flooding duration treatments, respectively, the number of new stems per pot and the height (cm) of five haphazardly selected stems per pot were measured. Three of the five selected stems were cut, and the fresh and oven‐dried (65°C for 72 h) weights of the aboveground biomass (stem and leaves) were measured and used to determine mean biomass per stem and dry matter content (DMC = dry weight/fresh weight × 100%). The aboveground biomass of the total population of *C. cinerascens* per pot was estimated based on the mean stem biomass × number of stems per pot.

During the following spring season (early April), the number of flowering stems per pot was recorded at the peak of the flowering phase, and shortly thereafter, at the peak of biomass production (1 month later for all flooding treatments), the number of stems per pot, the height (cm) of five haphazardly selected stems per pot, and the relative cover of *C. cinerascens* were measured. In addition, all the aboveground biomass of each pot was collected, oven‐dried at 65°C for 72 h, and weighed. Three individuals of *C. cinerascens* were used in order to determine the mean biomass per stem (g) and DMC (%).

### 2.5 Statistical Analysis

Statistical analyses were performed using the R software 3.2.3. Considering that two replicates of each treatment were put in the same tank (block), we used linear mixed‐effect models (package *nlme*) with replicate nested into block (i.e., two replicates per treatment per tank) as a random factor followed by a post hoc Tukey test (package *multcomp*) to test the effects of the flooding treatments (6 scenarios) on growth and reproductive traits of *C. cinerascens* . Significance was evaluated at *p* < 0.05 in all cases. Data were log‐transformed when necessary to meet the conditions of normality and homoscedasticity.

## 3 Results

### 3.1 Plant Responses During and at the End of the Flooding Period

**FIGURE 2:**
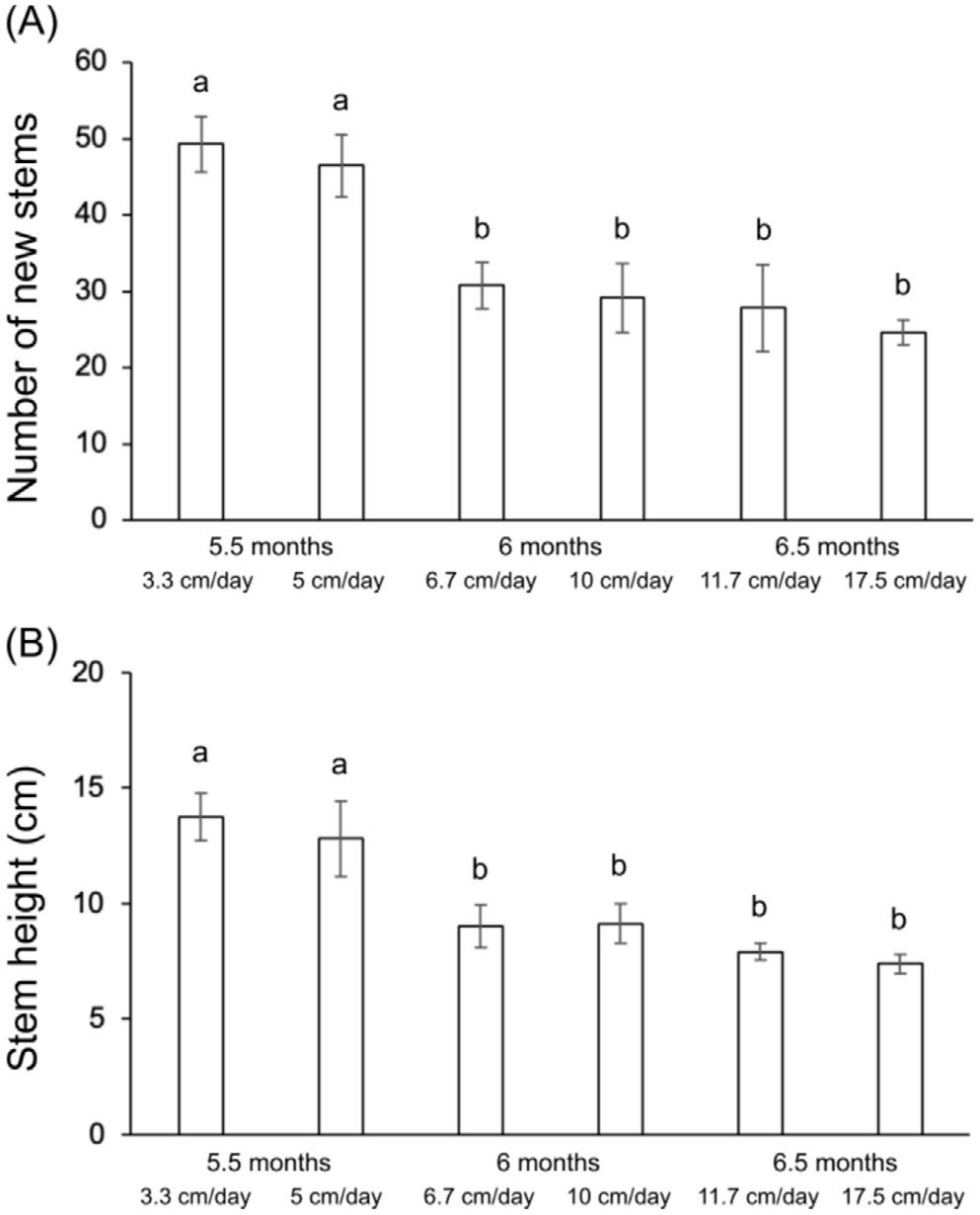
Number (A) and height (B) of new stems of *Carex cinerascens* during the flooding period (i.e., 1 month after the beginning of flooding) for the six flooding treatments. During the flooding period, the six flooding treatments only differ by their submergence rate. Values are mean ± SE (*n* = 6). Different letters indicate significant differences between treatments with a > b > c (Tukey tests).

After 1 month of flooding, the flooding treatments only differed by their submergence rate and we found that the number of new stems that developed during the flooding period (Figure 2A, Table 1), as well as the stem height (Figure 2B, Table 1), was strongly reduced by about 40% and 30%, respectively, for submergence rates higher than 5 cm/day.

**TABLE 1:** Outputs of the linear mixed‐effect models testing the effects of the six flooding treatments on growth and reproductive traits of *Carex cinerascens*.

	Treatment	
	*F*_5,25_	*p*
Before flooding		
Number of stems	0.08	0.9947
During flooding		
Number of new stems	8.05	**< 0.001**
Stem height (cm)	8.75	**< 0.001**
End of flooding		
Number of new stems	88.56	**< 0.001**
Stem height (cm)	20.92	**< 0.001**
Number of old stems	10.86	**< 0.001**
Autumn after flooding		
Number of stems	7.69	**< 0.001**
Stem height (cm)	3.63	**0.0131**
Dry matter content (%)	9.83	**< 0.001**
Individual biomass (g.plant^−1^)	1.37	0.2676
Community biomass (g.pot^−1^)	5.76	**0.0011**
Spring after flooding		
Stem number	4.73	**0.0035**
Stem height (cm)	1.12	0.3758
Dry matter content (%)	3.44	**0.0167**
Individual biomass (g.plant^−1^)	0.75	0.5933
Community biomass (g.pot^−1^)	3.50	**0.0156**
Plant cover (%)	23.81	**< 0.001**
Inflorescence number	18.14	**< 0.001**

*Note: F*‐values and associated *p*‐values are indicated and in bold when significant.

**FIGURE 3:**
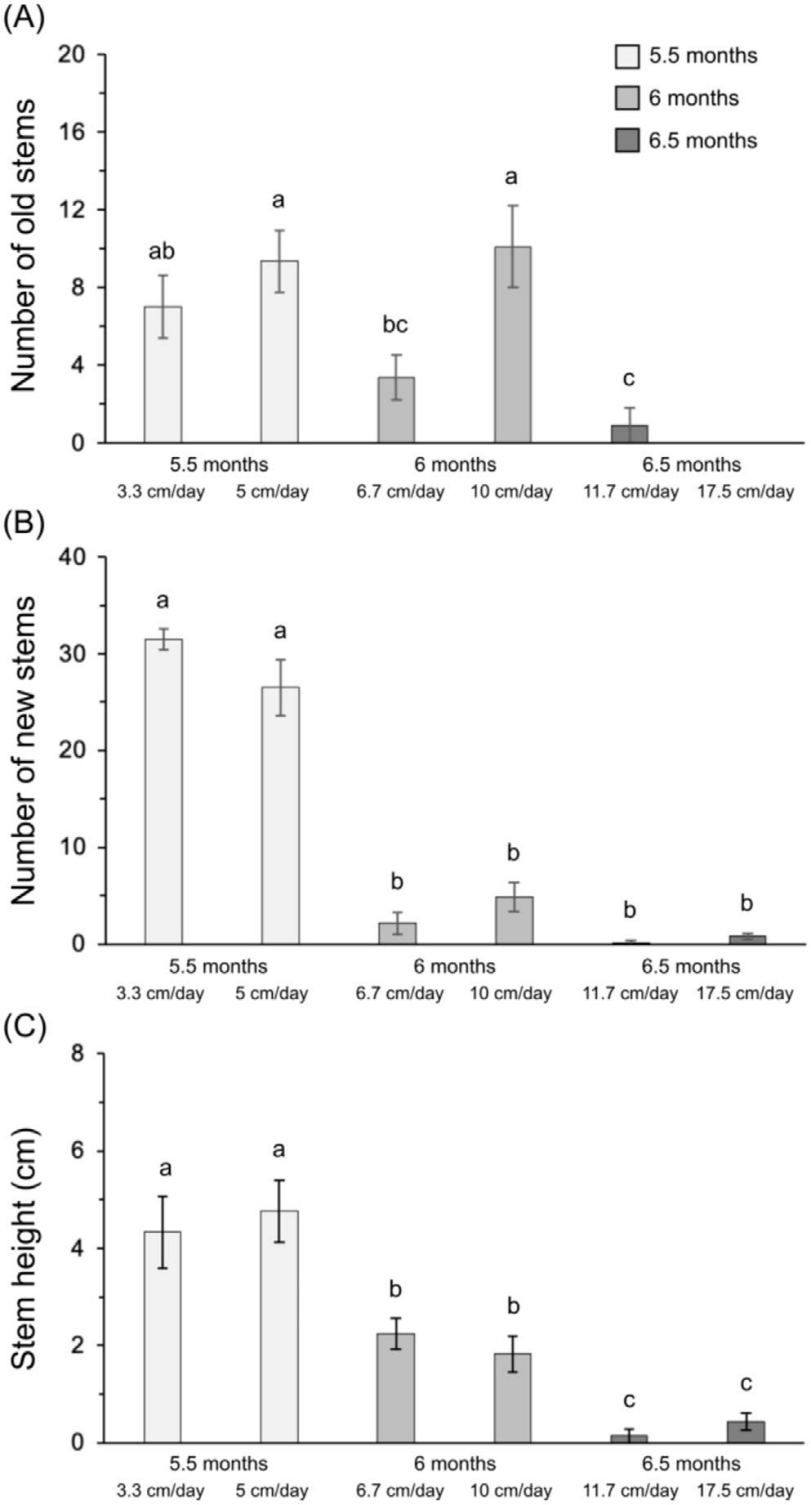
Number of old stems (A), number of new stems (B) and height of new stems (C) of *Carex cinerascens* at the end of the flooding period for the six flooding treatments. Values are mean ± SE (*n* = 6). Different letters indicate significant differences between flooding treatments with a > b > c (Tukey tests).

At the end of the flooding treatment, we observed a significant reduction in the average number of old stems in the 6.5‐month flooding treatments (0.4 stems) compared to the 5.5‐month (8.3 stems) and 6‐month (6.7 stems) flooding treatments (Figure 3A, Table 1). The number of new stems also strongly decreased in the 6‐month and 6.5‐month flooding treatments (respectively 3.5 and 1 stems on average) compared to the 5.5‐month flooding treatment (29 stems on average, Figure 3B). Finally, the stem height was also strongly reduced by the increase in flooding duration, ranging from 4.5 cm after 5.5 months of flooding, 2 cm after 6 months, to 0.3 cm after 6.5 months (Figure 3C). Overall, the submergence rate within each flooding duration treatment had no impact on plant traits during or at the end of the flooding (Figures 2, 3).

### 3.2 Plant Responses During Autumn and Spring Following the Flooding Events

**FIGURE 4:**
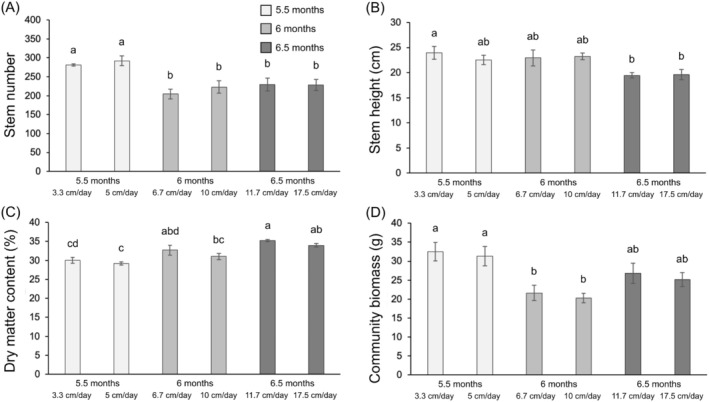
Stem number (A), stem height (B), dry matter content (C) and community aboveground biomass (D) of *C. cinerascens* at the peak of biomass production in autumn for the six flooding treatments. Values are mean ± SE (*n* = 6). Different letters indicate significant differences between flooding treatments with a > b > c (Tukey tests).

In the post‐flooding autumn period, the flooding treatments had significant effects on all the measured plant parameters, except on individual plant biomass (Table 1), but there was no significant difference in plant parameters between the two submergence rates within the same flooding duration (Figure 4). The average number of *C. cinerascens* stems was 29% lower in the 6‐month (214 stems) and 6.5‐month (229 stems) flooding treatments compared to the 5.5‐month flooding treatments (287 stems, Figure 4A). The stem height of *C. cinerascens* was slightly reduced in the 6.5‐month flooding treatments compared to the 5.5‐month flooding treatments (19.5 cm and 23.3 cm, respectively, Figure 4B). The dry matter content of *C. cinerascens* showed an increasing trend with the increasing flooding duration, with significantly higher values in the 6.5‐month flooding treatment (+17%) compared to the 5.5‐month flooding treatments (Figure 4C). The community aboveground biomass was significantly higher by 34% in the 5.5‐month flooding treatments (31.9 g.pot^−1^ on average) compared to the 6‐month flooding treatments (25.9 g.pot^−1^) with an intermediate value in the 6.5‐month (20.9 g.pot^−1^) flooding treatments (Figure 4D).

**FIGURE 5:**
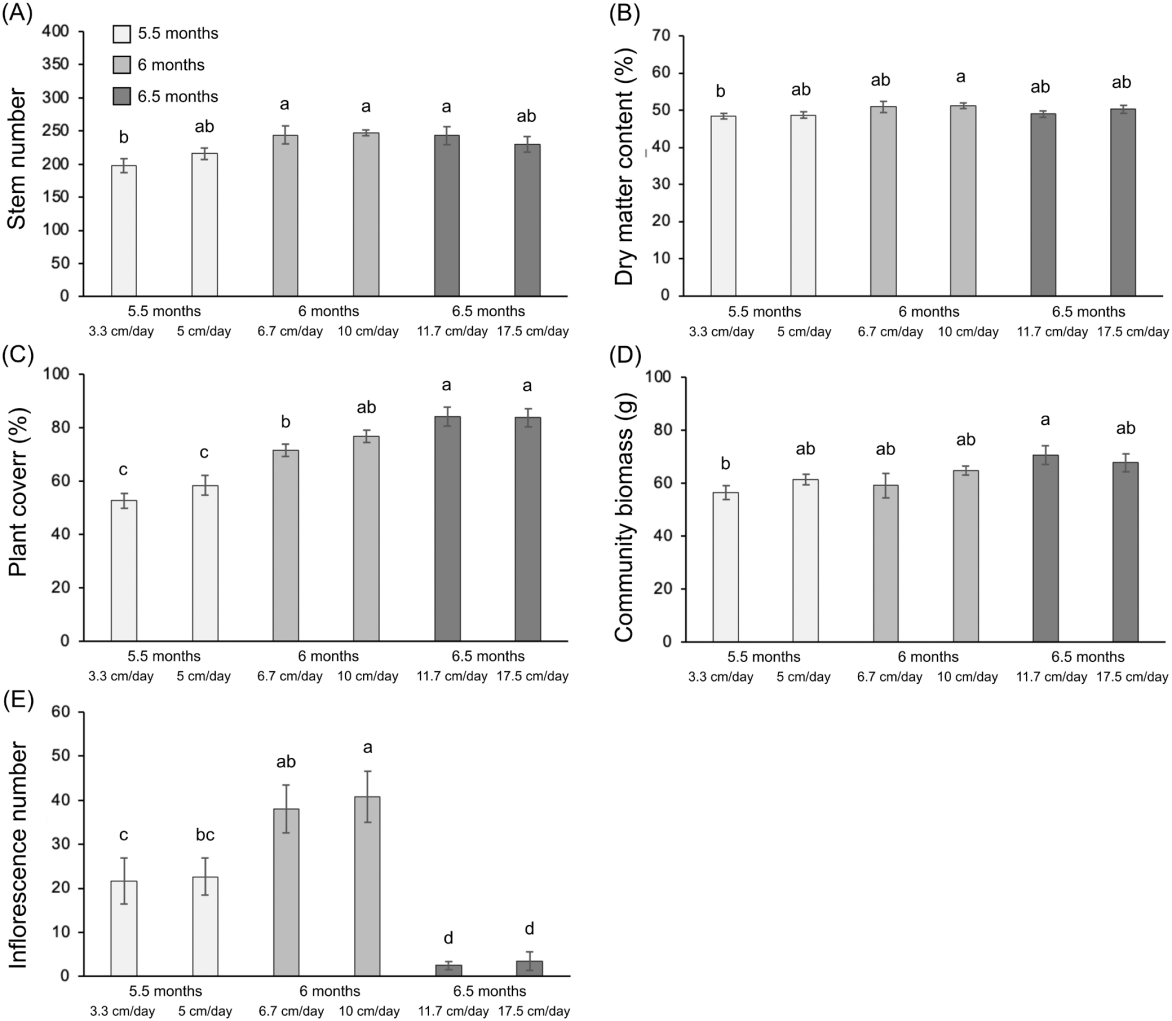
Stem number (A), dry matter content (B), relative plant cover (C), community aboveground biomass (D), and number of inflorescence (E) of *C. cinerascens* in spring following the flooding event according to the six flooding treatments. Values are mean ± SE (*n* = 6). Different letters indicate significant differences among flooding treatments with a > b > c (Tukey tests).

In the post‐flooding spring period, all the parameters measured on *C. cinerascens* , except for stem height and individual plant biomass, were still significantly affected by the flooding treatments (Table 1). However, the two submergence rates within the same flooding duration did not have any significant effect on the plant parameters (Figure 5). *C. cinerascens* produced 14% more stems in the 6‐month and 6.5‐month flooding treatments (respectively, 245 and 237 stems on average) compared to the 5.5‐month flooding treatments (207 stems, Figure 5A). The dry matter content of *C. cinerascens* did not vary much among the different flooding treatments and was 49.7% on average (Figure 5B). The plant cover of *C. cinerascens* significantly increased with increasing flooding duration, from 55% in the 5.5‐month, 74% in the 6‐month to 84% in the 6.5‐month flooding treatments (Figure 5C). The community aboveground biomass showed an increasing trend with increasing flooding duration (from 58.9 g.pot^−1^ in 5.5 months to 69.1 g.pot^−1^ in 6.5 months of flooding, Figure 5D). Finally, strong differences in the number of flowering stems were observed among the different flooding treatments (Table 1). Indeed, an average of 39 flowering stems per pot was observed in the 6‐month, 22 in the 5.5‐month and 3 in the 6.5‐month flooding treatments (Figure 5E).

## 4 Discussion

### 4.1 Plant Responses During and at the End of the Flooding Events

As we expected, the submergence rate, ranging from 3.3 to 17.5 cm per day, impacted the growth traits of *C. cinerascens* during the flooding. We observed a significant decrease in the number of new stems and stem height for submergence rates higher than 5 cm per day, which is consistent with previous studies performed in similar wetland systems (Yao et al. 2021; Hu et al. 2022). Since the submergence rate directly controls underwater light availability (Vervuren et al. 2003; Serrano et al. 2014), the pots with a submergence rate lower than 5 cm per day received much more light, thus improving both recruitment and growth of young plants (Clevering et al. 1996; Li et al. 2011).

At the end of the floodings, *C. cinerascens* produced more new stems with greater height in the 5.5‐month flooding treatments compared to longer floods. Longer flooding duration may reduce the growth of wetland plant species due to lower photosynthesis and stomatal conductance rates (Mills et al. 2011; Zhao et al. 2021). With a faster submergence rate and longer flooding duration in the 6‐month and 6.5‐month flooding treatments, a lower availability of photosynthetic active radiation caused a strong reduction in new stem development and growth rates. Furthermore, a flooding duration of 6.5 months induced a faster decay of old stems that almost completely disappeared at the end of the flooding, likely due to a faster decomposition rate of submerged plant materials (Zhang et al. 2019). In contrast to our second hypothesis, the submergence rate did not amplify the negative effects of flooding duration within each flooding duration treatment. These findings suggest that the submergence rate might only influence *C. cinerascens* growth at the beginning of the flood, as described above, and that flooding duration has a much stronger impact than submergence rate at the end of flooding.

### 4.2 Post‐Flooding Plant Responses During the Subsequent Autumn Growing Season

As we hypothesized, the shortest summer flooding duration had lower negative impacts on *C. cinerascens* growth during the autumn growing season, but submergence rate manipulation within the same flooding duration had no additional effect. We reported a higher number of *C. cinerascens* stems and higher stem height in the 5.5‐month flooding treatments compared to the four other treatments. Since the 5.5‐month floods ended earlier, the plant community was exposed to better climatic conditions (light and temperature), which promoted photosynthesis and subsequently plant growth. Similar to Fu et al. (2018), who reported that plants exhibited decreasing leaf dry matter content during the autumn growing season with decreasing summer flooding in the Poyang Lake wetland, we also found an increase in dry matter content (DMC) with increasing summer flooding duration in the autumn growing season. Lower DMC values in the 5.5‐month flooding treatments in the present study may be associated with higher investment in assimilatory tissue (i.e., the mesophyll) (Wright et al. 2004; Kazakou et al. 2006). Thus, the range of DMC values across the six flooding scenarios likely reflects a trade‐off between higher energy investment in plant growth (i.e., enlargement of cell walls and higher photosynthesis) under lower flooding stress and higher investment in plant maintenance (i.e., increase in the rigidity of cell walls) under higher flooding stress.

In line with van Eck et al. (2004), Guan et al. (2014) and Campbell et al. (2016), we found a general decrease in aboveground biomass with increasing flooding duration. Since the different flooding treatments had no impact on individual stem biomass, the reduction in community biomass in the 6‐month and 6.5‐month treatments compared to the 5.5‐month flooding treatments was mostly explained by the lower stem number of *C. cinarescens*. The community biomass in the pots experiencing the 6.5‐month flooding treatments was lower but not significantly different from that of the 5.5‐month flooding treatments, which might be related to higher nutrient availability under longer flooding duration (Wright et al. 2015; Lan et al. 2021) and growth overcompensation (i.e., higher recovery rate) after stronger flooding stress (Mollard et al. 2022). Interestingly, the peak of biomass production in the 6‐month and 6.5‐month flooding treatments occurred, respectively, 1 and 2 months later than in the 5.5‐month flooding treatments, while their flooding durations were, respectively, 0.5 and 1 month longer than the 5.5‐month flooding treatments. Thus, we reported a delay in the peak of biomass production in autumn with increasing flooding duration corresponding to twice the difference in flooding duration among treatments. Such findings are in agreement with Guan et al. (2014) who reported a delay in peak biomass of *Carex* meadows in response to delayed flood recession. In addition, Jing et al. (2017) highlighted that the time of flood recession exerted an important control on the distribution of *Carex* meadows in Dongting Lake (China). Therefore, the flooding duration, including the flood stress and the time of recession, really matters for the regrowth of wetland plants in the following growing season, with strong impacts on the stem number and thus on the population growth.

### 4.3 Post‐Flooding Plant Responses During the Subsequent Spring Growing Season

Contrary to our expectation, the effects of the summer flooding events persisted into the following spring. Surprisingly, we reported an opposite trend of *C. cinerascens* growth traits response to flooding treatments in spring compared to autumn. Indeed, stem number, plant cover, and community biomass were lower in the 5.5‐month flooding treatments compared to the longer flooding durations. These findings suggest a trade‐off between recovery and resilience to flooding, depending on flooding duration, with *C. cinerascens* ‐dominated communities showing higher recovery (autumn) but lower resilience (spring) to the 5.5‐month flooding treatments and lower recovery but higher resilience to the 6‐month and 6.5‐month flooding treatments (Mollard et al. 2022).

The number of inflorescences per pot was higher in the 6‐month flooding treatments and drastically lower in the 6.5‐month flooding treatments compared to the 5.5‐month flooding treatments. As highlighted by Chen et al. (2015) for *Carex brevicuspis* C.B. Clarke, higher flooding stress induces higher production of reproductive ramets as a survival mechanism, which is in line with the increase in inflorescence of *C. cinerascens* observed in the 6‐month compared to the 5.5‐month flooding treatments. In the 6.5‐month flooding treatments, the very low number of inflorescences recorded in spring is likely related to the higher investment in biomass production (i.e., community biomass), and the shorter growing time and delayed peak of biomass production observed in autumn, which means less energy accumulation compared to other treatments. This limited the energy allocation to flower production in the following spring in the 6.5‐month flooding treatments (Chiariello and Gulmon 1991). Overall, our findings suggest a trade‐off between resource allocation to biomass production in autumn and resource allocation to sexual reproduction in spring, which is mediated by flooding duration. These results have strong implications for the dynamics of the *C. cinerascens* population, since long floodings (6.5 months) would reduce sexual reproduction (i.e., almost no flowers in our study) and thus seed dispersal. In the long term, this can lead to a reduction in the population size of *C. cinerascens* , especially in the case of recurrent prolonged flooding events.

## 5 Conclusions

We observed that the submergence rate only impacted the growth traits of *C. cinerascens* during flooding, whereas it had limited impacts at the end and during the post‐flooding periods (i.e., recovery in autumn and resilience in spring). Flooding duration was the main factor affecting the growth traits of *C. cinerascens* from the end of the flooding treatments till the following spring. Shorter flooding duration generally induced lower negative effects on plant growth traits at the end of the flooding and during the recovery phase in autumn, whereas longer flooding caused a strong delay in the peak of biomass production. Our findings also highlighted a trade‐off between growth recovery (autumn) and resilience (spring) with *C. cinerascens* ‐dominated communities showing a stronger recovery but lower resilience to flooding in the shorter flooding duration treatment (5.5 months), whereas an opposite trend was observed in the longer flooding duration treatments (6‐month and 6.5‐months). Similarly, we demonstrated a trade‐off between resource allocation to biomass production in autumn and resource allocation to sexual reproduction in spring, with higher biomass production in autumn and lower sexual reproduction in spring for the shortest flooding treatment (5.5‐months) and lower biomass production in autumn and higher sexual reproduction in spring in the intermediate flooding treatment (6‐months). We also observed that the longest flooding treatments almost completely suppressed sexual reproduction of *C. cinerascens* by modifying plant phenology (i.e., delay in the peak of biomass production) and resource allocation (i.e., investment in growth rather than in flowers). Overall, the results from our study shed light on the legacy effects of flooding duration on *C. cinerascens* growth and reproductive traits while providing important data to predict changes in *C. cinerascens* population dynamics under forecasted climate change‐induced flooding. While previous studies generally focused on the impact of flooding on a single growing period (e.g., You et al. 2015; Dai et al. 2020; Liu et al. 2023), future research should investigate the influence of flooding along the whole plant life cycle, especially in ecosystems highly affected by hydrological processes. Long‐term studies are also needed to fully understand the implications of flooding duration on the fitness and population dynamics of *C. cinerascens*.

## Author Contributions

**Wenlan Feng:** conceptualization (equal), data curation (equal), formal analysis (equal), methodology (equal), writing – original draft (lead), writing – review and editing (equal). **Pierre Mariotte:** data curation (equal), formal analysis (equal), methodology (equal), supervision (equal), writing – original draft (equal), writing – review and editing (equal). **Ligang Xu:** conceptualization (equal), funding acquisition (equal), supervision (equal), writing – review and editing (equal). **Luca Bragazza:** supervision (equal), writing – review and editing (equal). **Alexandre Buttler:** supervision (equal), writing – review and editing (equal). **Junxiang Cheng:** conceptualization (equal), funding acquisition (equal), supervision (equal), writing – review and editing (equal). **Mathieu Santonja:** data curation (equal), formal analysis (equal), methodology (equal), supervision (equal), writing – original draft (equal), writing – review and editing (equal).

## Conflicts of Interest

The authors declare no conflicts of interest.

## Supporting information


Appendix S1.

## Data Availability

Data and code were available as Supporting information for the reviewers and editors during the review process. There are now available in the Dryad Digital Repository: https://doi.org/10.5061/dryad.q573n5tv7.

## References

[ece371395-bib-0001] Altenfelder, S. , M. Schmitz , P. Poschlod , J. Kollman , and H. Albrecht . 2016. “Managing Plant Species Diversity Under Fluctuating Wetland Conditions: The Case of Temporarily Flooded Depressions.” Wetlands Ecology and Management 24: 597–608. 10.1007/s11273-016-9490-2.

[ece371395-bib-0002] Bailey‐Serres, J. , and L. A. C. J. Voesenek . 2008. “Flooding Stress: Acclimations and Genetic Diversity.” Annual Review of Plant Biology 59: 313–339. 10.1146/annurev.arplant.59.032607.092752. 18444902

[ece371395-bib-0003] Baladron, A. , M. D. Bejarano , and I. Boavida . 2023. “Why Do Plants Respond Differently to Hydropeaking Disturbance? A Functional Approach.” Ecological Indicators 150: 110237. 10.1016/j.ecolind.2023.11023.

[ece371395-bib-0006] Campbell, D. , P. A. Keddy , M. Broussard , and T. B. McFalls‐Smith . 2016. “Small Changes in Flooding Have Large Consequences: Experimental Data From Ten Wetland Plants.” Wetlands 36: 457–466. 10.1007/s13157-016-0754-7.

[ece371395-bib-0007] Casanova, M. T. , and M. A. Brock . 2000. “How Do Depth, Duration and Frequency of Flooding Influence the Establishment of Wetland Plant Communities?” Plant Ecology 147: 237–250. 10.1023/A:1009875226637.

[ece371395-bib-0008] Chen, F. Q. , and Z. Q. Xie . 2009. “Survival and Growth Responses of *Myricaria laxiflora* Seedlings to Summer Flooding.” Aquatic Botany 90: 333–338. 10.1016/j.aquabot.2008.12.006.

[ece371395-bib-0009] Chen, H. J. , M. F. Zamorano , and D. Ivanoff . 2013. “Effect of Deep Flooding on Nutrients and Non‐Structural Carbohydrates of Mature *Typha domingensis* and Its Post‐Flooding Recovery.” Ecological Engineering 53: 267–274. 10.1016/j.ecoleng.2012.12.056.

[ece371395-bib-0011] Chen, X. S. , Y. F. Li , Y. H. Xie , Z. M. Deng , X. Li , and F. Li . 2015. “Trade‐Off Between Allocation to Reproductive Ramets and Rhizome Buds in *Carex brevicuspis* Populations Along a Small‐Scale Elevational Gradient.” Scientific Reports 5: 12688. 10.1038/srep12688. 26228352 PMC4521143

[ece371395-bib-0101] Chiariello, N. R. , and S. L. Gulmon . 1991. “Stress effects on plant reproduction.” In Response of plants to multiple stresses, edited by H. A. Mooney , W. E. Winner , and E. J. Pell , 161–188. Academic Press.

[ece371395-bib-0012] Clevering, O. A. , C. W. P. M. Blom , and W. V. Van Vierssen . 1996. “Growth and Morphology of *Scirpus lacustris* and *S. maritimus* Seedlings as Affected by Water Level and Light Availability.” Functional Ecology 10: 289–296. 10.2307/2389855.

[ece371395-bib-0013] Cooling, M. P. , G. G. Ganf , and K. F. Walker . 2001. “Leaf Recruitment and Elongation: An Adaptive Response to Flooding in *Villarsia reniformis* .” Aquatic Botany 70: 281–294. 10.1016/S0304-3770(01)00153-X.

[ece371395-bib-0014] Cui, X. H. , Y. Zhong , and J. K. Chen . 2000. “Influence of a Catastrophic Flood on Densities and Biomasses of Three Plant Species in Poyang Lake, China.” Journal of Freshwater Ecology 15, no. 4: 537–541. 10.1080/02705060.2000.9663776.

[ece371395-bib-0015] Dai, X. , Z. Yu , G. Yang , and R. Wan . 2020. “Role of Flooding Patterns in the Biomass Production of Vegetation in a Typical Herbaceous Wetland, Poyang Lake Wetland, China.” Frontiers in Plant Science 11: 521358. 10.3389/fpls.2020.521358. 33178232 PMC7596249

[ece371395-bib-0016] Dawson, T. P. , P. M. Berry , and E. Kampa . 2003. “Climate Change Impacts on Freshwater Wetland Habitats.” Journal for Nature Conservation 11: 25–30. 10.1078/1617-1381-00031.

[ece371395-bib-0017] Deegan, B. M. , S. D. White , and G. G. Ganf . 2012. “Nutrients and Water Level Fluctuations: A Study of Three Aquatic Plants.” River Research and Applications 28: 359–368. 10.1002/rra.1461.

[ece371395-bib-0018] Erwin, K. L. , and R. C. Gardner . 2009. “Wetlands and Global Climate Change: The Role of Wetland Restoration in a Changing World.” Wetlands Ecology and Management 17: 71. 10.1007/s11273-008-9119-1.

[ece371395-bib-0019] Feng, W. , P. Mariotte , L. Xu , et al. 2020. “Seasonal Variability of Groundwater Level Effects on the Growth of *Carex cinerascens* in Lake Wetlands.” Ecology and Evolution 10: 517–526. 10.1002/ece3.5926. 31988739 PMC6972833

[ece371395-bib-0020] Feng, W. , M. Santonja , L. Bragazza , and A. Buttler . 2020. “Shift in Plant‐Soil Interactions Along a Lakeshore Hydrological Gradient.” Science of the Total Environment 742: 140254. 10.1016/j.scitotenv.2020.140254. 32721708

[ece371395-bib-0022] Fu, H. , Q. Lou , T. T. Dai , et al. 2018. “Hydrological Gradients and Functional Diversity of Plants Drive Ecosystem Processes in Poyang Lake Wetland.” Ecohydrology 11: e1950. 10.1002/eco.1950.

[ece371395-bib-0023] Garssen, A. G. , A. Baattruppedersen , T. Riis , et al. 2017. “Effects of Increased Flooding on Riparian Vegetation: Field Experiments Simulating Climate Change Along Five European Lowland Streams.” Global Change Biology 23: 3052–3063. 10.1111/gcb.13687. 28295947

[ece371395-bib-0024] Garssen, A. G. , A. Baattruppedersen , L. A. Voesenek , J. T. Verhoeven , and M. B. Soons . 2015. “Riparian Plant Community Responses to Increased Flooding: A Meta‐Analysis.” Global Change Biology 21: 2881–2890. 10.1111/gcb.12921. 25752818

[ece371395-bib-0025] Guan, L. , L. Wen , D. D. Feng , H. Zhang , and G. C. Lei . 2014. “Delayed Flood Recession in Central Yangtze Floodplains Can Cause Significant Food Shortages for Wintering Geese: Results of Inundation Experiment.” Environmental Management 54: 1331–1341. 10.1007/s00267-014-0350-7. 25164981

[ece371395-bib-0026] Guo, H. , Q. Hu , Q. Zhang , and S. Feng . 2012. “Effects of the Three Gorges Dam on Yangtze River Flow and River Interaction With Poyang Lake, China: 2003‐2008.” Journal of Hydrology 416‐417: 19–27.

[ece371395-bib-0027] Hu, C. , C. H. Xu , G. Hu , Z. H. Zhang , F. Li , and Y. H. Xie . 2022. “Effect of Water Level and Submergence Time on Leaf Growth, Stoichiometry and Homeostasis of Carex Brevicuspis.” Applied Ecology and Environmental Research 21, no. 1: 623–635. 10.15666/aeer/2101_623635.

[ece371395-bib-0028] Hu, J. Y. , Y. H. Xie , Y. Tang , F. Li , and Y. A. Zou . 2018. “Changes of Vegetation Distribution in the East Dongting Lake After the Operation of the Three Gorges Dam, China.” Frontiers in Plant Science 9: 582. 10.3389/fpls.2018.00582. 29765388 PMC5938568

[ece371395-bib-0029] Hu, Y. , J. Huang , Y. Du , P. Han , and W. Huang . 2015. “Monitoring Spatial and Temporal Dynamics of Flood Regimes and Their Relation to Wetland Landscape Patterns in Dongting Lake From MODIS Time‐Series Imagery.” Remote Sensing 7: 7494–7520. 10.3390/rs70607494.

[ece371395-bib-0030] Huang, W. , T. Hu , J. Mao , et al. 2022. “Hydrological Drivers for the Spatial Distribution of Wetland Herbaceous Communities in Poyang Lake.” Remote Sensing 14, no. 19: 4870. 10.3390/rs14194870.

[ece371395-bib-0100] Jing, L. , C. Lu , Y. Xia , et al. 2017. “Effects of hydrological regime on development of Carex wet meadows in East Dongting Lake, a Ramsar Wetland for wintering waterbirds.” Scientific Reports 7, no. 1: 41761. 10.1038/srep41761. 28165508 PMC5292947

[ece371395-bib-0031] Kazakou, E. , D. Vile , C. G. Shipley , and E. Garnier . 2006. “Co‐Variations in Litter Decomposition, Leaf Traits and Plant Growth in Species From a Mediterranean Old‐Field Succession.” Functional Ecology 20: 21–30. 10.1111/j.1365-2435.2006.01080.x.

[ece371395-bib-0032] Lai, X. , D. Shankman , C. Huber , H. Yesou , Q. Huang , and J. Jiang . 2014. “Sand Mining and Increasing Poyang Lake's Discharge Ability: A Reassessment of Causes for Lake Decline in China.” Journal of Hydrology 519: 1698–1706. 10.1016/j.jhydrol.2014.09.058.

[ece371395-bib-0033] Lan, Z. , Y. Chen , R. Shen , et al. 2021. “Effects of Flooding Duration on Wetland Plant Biomass: The Importance of Soil Nutrients and Season.” 66: 211–222. 10.1111/fwb.13630.

[ece371395-bib-0034] Langerwisch, F. , S. Rost , D. Gerten , B. Poulter , A. Ramming , and W. Cramer . 2013. “Potential Effects of Climate Change on Inundation Patterns in the Amazon Basin.” Hydrology and Earth System Sciences Discussions 9: 261–300. 10.5194/hess-17-2247-2013.

[ece371395-bib-0035] Li, B. , G. S. Yang , and R. R. Wan . 2023. “Reassessment of the Declines in the Largest Freshwater Lake in China (Poyang Lake): Uneven Trends, Risks and Underlying Causes.” Journal of Environmental Management 342: 118157. 10.1016/j.jenvman.2023.118157. 37196623

[ece371395-bib-0036] Li, F. , Y. Li , H. Qin , and Y. H. Xie . 2011. “Plant Distribution Can Be Reflected by the Different Growth and Morphological Responses to Water Level and Shade in Two Emergent Macrophyte Seedlings in the Sanjiang Plain.” Aquatic Ecology 45: 89–97. 10.1007/s10452-010-9334-8.

[ece371395-bib-0037] Li, W. , X. Wang , L. He , Y. Liu , and G. Ge . 2018. “The Responses of Growth and Vegetative Reproduction of Wetland Plants in Poyang Lake to Duration of Submergence.” Acta Ecologica Sinica 38, no. 22: 8176–8183.

[ece371395-bib-0038] Li, Y. , Q. Zhang , Z. Tan , and J. Yao . 2020. “On the Hydrodynamic Behavior of Floodplain Vegetation in a Flood‐Pulse‐Influenced River‐Lake System (Poyang Lake, China).” Journal of Hydrology 585: 124852. 10.1016/j.jhydrol.2020.124852.

[ece371395-bib-0039] Liu, Y. , J. Li , Y. Liu , et al. 2023. “Interactive Effects of Flooding Duration and Sediment Texture on the Growth and Adaptation of Three Plant Species in the Poyang Lake Wetland.” Biology‐Basel 12, no. 7: 944. 10.3390/biology12070944. 37508375 PMC10376433

[ece371395-bib-0040] Lunt, I. D. , A. Jansen , D. L. Binns , and A. Sheppard . 2012. “Effects of Flood Timing and Livestock Grazing on Exotic Annual Plants in Riverine Floodplains.” Journal of Applied Ecology 49, no. 9: 1131–1139. 10.1111/j.1365-2664.2012.02176.x.

[ece371395-bib-0043] Mills, M. C. , B. Baldwin , and G. N. Ervin . 2011. “Evaluating Physiological and Growth Responses of *Arundinaria* Species to Inundation.” Castanea 76: 395–409. 10.2307/41416186.

[ece371395-bib-0044] Mollard, F. P. , C. E. Di Bella , M. B. Loguzzo , A. A. Grimoldi , and G. G. Striker . 2022. “High Recovery From Either Waterlogging or Drought Overrides Any Beneficial Acclimation of *Chloris gayana* Facing a Subsequent Round of Stress.” Plants 11: 2699. 36297722 10.3390/plants11202699 PMC9610420

[ece371395-bib-0045] Mommer, L. , and E. J. W. Visser . 2005. “Underwater Photosynthesis in Flooded Terrestrial Plants: A Matter of Leaf Plasticity.” Annals of Botany 96: 581–589. 10.1093/aob/mci212. 16024559 PMC4247027

[ece371395-bib-0046] Neckles, H. A. , H. R. Murkin , and J. A. Cooper . 2010. “Influences of Seasonal Flooding on Macroinvertebrate Abundance in Wetland Habitats.” Freshwater Biology 23: 311–322. 10.1111/j.1365-2427.1990.tb00274.x.

[ece371395-bib-0047] Paillisson, J. M. , and L. Marion . 2011. “Water Level Fluctuations for Managing Excessive Plant Biomass in Shallow Lakes.” Ecological Engineering 37: 241–247. 10.1016/j.ecoleng.2010.11.017.

[ece371395-bib-0048] Pan, Y. , E. Cieraad , B. R. Clarkson , et al. 2020. “Drivers of Plant Traits That Allow Survival in Wetlands.” Functional Ecology 34: 956–967. 10.1111/1365-2435.13541.

[ece371395-bib-0049] Pezeshki, S. R. 2001. “Wetland Plant Responses to Soil Flooding.” Environmental and Experimental Botany 46: 299–312. 10.1016/S0098-8472(01)00107-1.

[ece371395-bib-0050] Raulings, E. J. , K. Morris , M. C. Roache , and P. I. Boon . 2010. “The Importance of Water Regimes Operating at Small Spatial Scales for the Diversity and Structure of Wetland Vegetation.” Freshwater Biology 55: 701–715. 10.1111/j.1365-2427.2009.02311.x.

[ece371395-bib-0051] Renofalt, B. M. , D. M. Merritt , and C. Nilsson . 2007. “Connecting Variation in Vegetation and Stream Flow: The Role of Geomorphic Context in Vegetation Response to Large Floods Along Boreal Rivers.” Journal of Applied Ecology 44: 147–157. 10.1111/j.1365-2664.2006.01223.x.

[ece371395-bib-0052] Sarneel, J. M. , M. M. Hefting , G. A. Kowalchuk , et al. 2019. “Alternative Transient States and Slow Plant Community Responses Alter Changed Flooding Regimes.” Global Change Biology 25: 1–10. 10.1111/gcb.14569. 30638293 PMC6849759

[ece371395-bib-0053] Serrano, O. , P. S. Lavery , M. Rozaimi , and M. A. Mateo . 2014. “Influence of Water Depth on the Carbon Sequestration Capacity of Seagrasses.” Global Biogeochemical Cycles 28: 950–961. 10.1002/2014GB004872.

[ece371395-bib-0054] Tan, Z. , H. Tao , J. Jiang , and Q. Zhang . 2015. “Influences of Climate Extremes on NDVI (Normalized Difference Vegetation Index) in the Poyang Lake Basin, China.” Wetlands 35: 1033–1042. 10.1007/s13157-015-0692-9.

[ece371395-bib-0055] van Eck, W. H. J. M. , H. M. van de Steeg , C. W. P. M. Blom , and H. de Kroon . 2004. “Is Tolerance to Summer Flooding Correlated With Distribution Patterns in River Floodplains? A Comparative Study of 20 Terrestrial Grassland Species.” Oikos 107: 393–405. 10.2307/3548222.

[ece371395-bib-0056] Vervuren, P. J. A. , C. W. P. M. Blom , and H. De Kroon . 2003. “Extreme Flooding Events on the Rhine and the Survival and Distribution of Riparian Plant Species.” Journal of Ecology 91: 135–146. 10.1046/j.1365-2745.2003.00749.x.

[ece371395-bib-0057] Visser, E. J. W. , G. M. Bögemann , H. M. Van de Steeg , R. Pierik , and C. Blom . 2000. “Flooding Tolerance of *Carex* Species in Relation to Field Distribution and Aerenchyma Formation.” New Phytologist 148, no. 1: 93–103. 10.1046/j.1469-8137.2000.00742.x. 33863031

[ece371395-bib-0058] Voesenek, L. A. C. J. , R. Sasidharan , E. J. W. Visser , and J. Bailey‐Serres . 2016. “Flooding Stress Signaling Through Perturbations in Oxygen, Ethylene, Nitric Oxide and Light.” New Phytologist 209: 39–43. 10.1111/nph.13775. 26625347

[ece371395-bib-0059] Wang, X. L. , L. G. Xu , R. R. Wan , and Y. W. Chen . 2016. “Seasonal Variations of Soil Microbial Biomass Within Two Typical Wetland Areas Along the Vegetation Gradient of Poyang Lake, China.” Catena 137: 483–493. 10.1016/j.catena.2015.10.020.

[ece371395-bib-0060] Webb, J. A. , E. M. Wallis , and M. J. Stewardson . 2012. “A Systematic Review of Published Evidence Linking Wetland Plants to Water Regime Components.” Aquatic Botany 103: 1–14. 10.1016/j.aquabot.2012.06.003.

[ece371395-bib-0061] Wei, H. , S. Cheng , F. He , W. Liang , and Z. Wu . 2014. “Growth Responses and Adaptations of the Emergent Macrophyte *Acorus calamus* Linn. To Different Water‐Level Fluctuations.” Journal of Freshwater Ecology 29: 101–116. 10.1080/02705060.2013.833142.

[ece371395-bib-0062] Wright, A. , A. Ebeling , H. de Kroon , et al. 2015. “Flooding Disturbances Increase Resource Availability and Productivity but Reduce Stability in Diverse Plant Communities.” Nature Communications 6: 6092. 10.1038/ncomms7092. 25600177

[ece371395-bib-0063] Wright, I. J. , P. B. Reich , M. Westoby , et al. 2004. “The Worldwide Leaf Economics Spectrum.” Nature 428: 821. 10.1038/nature02403. 15103368

[ece371395-bib-0064] Yang, Y. Y. , D. Yu , Y. K. Li , Y. H. Xie , and X. H. Geng . 2004. “Phenotypic Plasticity of Two Submersed Plants in Response to Flooding.” Journal of Freshwater Ecology 19: 69–76. 10.1080/02705060.2004.9664514.

[ece371395-bib-0066] Yao, X. , Y. Cao , G. Zheng , et al. 2021. “Ecological Adaptability and Population Growth Tolerance Characteristics of *Carex cinerascens* in Response to Water Level Changes in Poyang Lake, China.” Scientific Reports 11: 4887. 10.1038/s41598-021-84282-x. 33649457 PMC7921597

[ece371395-bib-0067] You, H. , L. Xu , G. Liu , X. Wang , Y. Wu , and J. Jiang . 2015. “Effects of Inter‐Annual Water Level Fluctuations on Vegetation Evolution in Typical Wetlands of Poyang Lake, China.” Wetlands 35: 931–943. 10.1007/s13157-015-0684-9.

[ece371395-bib-0068] Yu, L. , and D. Yu . 2011. “Differential Responses of the Floating‐Leaved Aquatic Plant *Nymphoides peltata* to Gradual Versus Rapid Increases in Water Levels.” Aquatic Botany 94: 71–76. 10.1016/j.aquabot.2010.11.004.

[ece371395-bib-0069] Yuan, S. B. , Z. D. Yang , X. Q. Liu , and H. Z. Wang . 2017. “Key Parameters of Water Level Fluctuations Determining the Distribution of Carex, in Shallow Lakes.” Wetlands 37: 1–10. 10.1007/s13157-017-0934-0.

[ece371395-bib-0070] Zedler, J. B. 2010. “How Frequent Storms Affect Wetland Vegetation: A Preview of Climate‐Change Impacts.” Frontiers in Ecology and the Environment 8: 540–547. 10.1890/090109.

[ece371395-bib-0071] Zhang, Q. , X. C. Ye , A. D. Werner , Y. L. Li , J. Yao , and X. H. Li . 2014. “An Investigation of Enhanced Recessions in Poyang Lake: Comparison of Yangtze River and Local Catchment Impacts.” Journal of Hydrology 517: 425–434. 10.1016/j.jhydrol.2014.05.051.

[ece371395-bib-0072] Zhang, Q. , G. Zhang , X. Yu , et al. 2019. “Effect of Ground Water Level on the Release of Carbon, Nitrogen and Phosphorus During Decomposition of *Carex cinerascens* Kükenth in the Typical Seasonal Floodplain in Dry Season.” Journal of Freshwater Ecology 34: 305–322.

[ece371395-bib-0073] Zhang, Z. T. , G. Q. Jin , H. W. Tang , S. Y. Zhang , D. Zhu , and J. Xu . 2022. “How Does the Three Gorges Dam Affect the Spatial and Temporal Variation of Water Levels in the Poyang Lake?” Journal of Hydrology 605: 127356. 10.1016/j.jhydrol.2021.127356.

[ece371395-bib-0074] Zhao, J. , S. L. Malone , C. L. Staudhamme , G. Starr , H. Hartmann , and S. F. Oberbauer . 2021. “Freshwater Wetland Plants Respond Nonlinearly to Inundation Over a Sustained Period.” American Journal of Botany 108, no. 10: 1917–1931. 10.1002/ajb2.1746. 34617586

[ece371395-bib-0075] Zheng, L. , X. Wang , D. Li , G. Xu , and Y. Guo . 2021. “Spatial Heterogeneity of Vegetation Extent and the Response to Water Level Fluctuations and Micro‐Topography in Poyang Lake, China.” Ecological Indicators 124: 107420. 10.1016/j.ecolind.2021.107420.

[ece371395-bib-0076] Zhou, Y. , X. Bai , and L. Ning . 2018. “Productivity of Carex Cinerascens Population and Its Response to Hydrological Conditions in the Poyang Lake Wetland.” Acta Ecologica Sinica 38, no. 14: 4953–4963.

