# Supplementary material for: Legacy Effects of Flooding Duration on Growth and Reproductive Traits of Carex cinerascens in the Poyang Lake Wetland
Source: Ecol Evol. 2025 May 6;15(5):e71395. doi: 10.1002/ece3.71395 (PMC12055085; doi:10.1002/ece3.71395)
Supplement: Supplementary file 1 — Appendix S1. [file ECE3-15-e71395-s001.docx]

**Supplementary Materials**

**Legacy effects of flooding duration on growth and reproductive traits of**

***Carex cinerascens* in the Poyang Lake wetland**

Wenlan Feng^1,2,3,4^, Pierre Mariotte^3,4,5^, Ligang Xu^1,6*^, Luca Bragazza^7^, Alexandre Buttler^3,4^, Junxiang Cheng^1,6^, Mathieu Santonja^3,4,8*^

**Addresses**

**1.** National Key Laboratory of Lake and Watershed Science for Water Security, Nanjing Institute of Geography and Limnology, Chinese Academy of Sciences, Nanjing, 210008, China.

**2.** State Key Laboratory of Soil and Sustainable Agriculture, Institute of Soil Science, Chinese Academy of Sciences, Nanjing 210008, China.

**3.** Ecole Polytechnique Fédérale de Lausanne (EPFL), School of Architecture, Civil and Environmental Engineering (ENAC), Laboratory of Ecological Systems (ECOS), Station 2, 1015 Lausanne, Switzerland.

**4.** Swiss Federal Institute for Forest, Snow and Landscape Research (WSL), Site Lausanne, Case postale 96, 1015 Lausanne, Switzerland.

**5.** Agroscope, Grazing Systems, 1725 Posieux, Switzerland.

**6.** University of Chinese Academy of Sciences, Beijing, 100049, China.

**7.** Agroscope, Field-Crop Systems and Plant Nutrition, 1260 Nyon, Switzerland.

**8.** Aix Marseille Univ, Avignon Univ, CNRS, IRD, IMBE, Marseille, France.

**Table S1.** Description of the flooding treatments according to flooding duration (months) and rate of submergence (cm/day). The faster rate of submergence within each flooding duration treatment was defined as a 50% increase of the slower submergence rate.

| Flooding treatment | Rate of submergence | | | | |
| --- | --- | --- | --- | --- | --- |
|  | Day 1 | Day 2 | Day 3 | Total | **Averaged** |
| 5.5 months with 10 cm/3days (slow) | 5 cm | 10 cm | -5 cm | 10 cm | **3.3 cm/day** |
| 5.5 months with 15 cm/3days (fast) | 5 cm | 10 cm | 0 cm | 15 cm | **5 cm/day** |
| 6.0 months with 20 cm/3days (slow) | 10 cm | 20 cm | -10 cm | 20 cm | **6.7 cm/day** |
| 6.0 months with 30 cm/3days (fast) | 10 cm | 20 cm | 0 cm | 30 cm | **10 cm/day** |
| 6.5 months with 35 cm/3days (slow) | 20 cm | 30 cm | -15 cm | 35 cm | **11.7 cm/day** |
| 6.5 months with 52.5 cm/3days (fast) | 20 cm | 32.5 cm | 0 cm | 52.5 cm | **17.5 cm/day** |

**Figure S1.** Relationship between submergence rate (cm day^-1^) and flooding duration (months) for the five flooding events in the Poyang Lake (China) that lasted longer than 5 months between 2004 and 2013 (10-year data obtained from the obtained from the Xingzi hydrologic station, Lushan city, Jiangxi Province, 300 m from the study site). Submergence rate was calculated as the speed of the water rising from 13 m to the highest water level in summer. Significant linear relationship is indicated with dotted line, adjusted R^2^ and associated *P*-values (** *P* < 0.01).

**
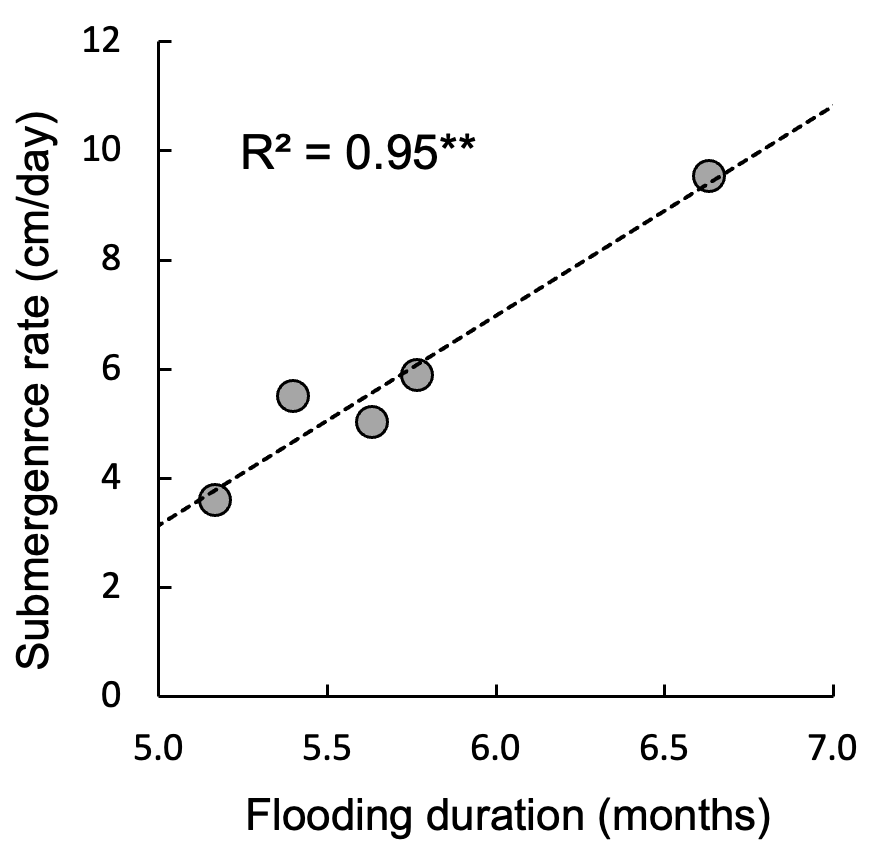
**

**Figure S2.** Schematic illustration of submergence rate treatments according to flooding duration over the first six days of the experiment (i.e. 2 cycles of 3 days, Supplementary Table S1 for more details). Similar cycles of three days were repeated till the pots touched the bottom of the tank.

**Figure S3.** Pictures of the pots in the tanks during the experiment with (a) tank covered by the sun shading net during flooding, (b) plant recovering in the autumn after the summer flooding and (c) plants recovering in the following spring after the summer flooding.
